# Relationship between exercise persistence and body image among overweight Chinese college students: the mediating role of fat talk and body self-esteem

**DOI:** 10.3389/fpsyg.2026.1824393

**Published:** 2026-06-04

**Authors:** Feng Yang, Jun Xiang, Jia Gao

**Affiliations:** 1 School of Physical Education and Health, Huaihua University, Huaihua, China; 2 School of Physical Education and Health, Zhaoqing University, Zhaoqing, China

**Keywords:** body image, body self-esteem, exercise persistence, fat talk, mediating effect, overweight college students

## Abstract

**Introduction:**

Exercise persistence is important for the well-being of overweight college students. However, the psychological mechanisms linking sustained exercise participation to body image are unexplored. This study examined the relationship between exercise persistence and body image among overweight Chinese college students, together with the mediating roles of fat talk and body self-esteem.

**Methods:**

Overall, 750 overweight college students from several Chinese universities completed the Exercise Persistence, Fat Talk, Body Self-Esteem, and Body Image Scales. Correlations and a chained mediation model explored the relationships among the study variables.

**Results:**

Exercise persistence was positively associated with body image and body self-esteem and negatively associated with fat talk. Fat talk was negatively related to body self-esteem and body image, whereas body self-esteem was positively associated with body image. Mediation analyses indicated that fat talk and body self-esteem mediated the relationship between exercise persistence and body image through both independent and sequential pathways.

**Conclusion:**

Sustained exercise engagement is linked to positive body image through reduced fat talk and enhanced body self-esteem. The findings suggest directions for future longitudinal and intervention research.

## 1 Introduction

Excess weight in young adults is a worldwide health concern. This is also seen in college students aged 18–24 in China. Body image refers to cognitive and emotional evaluations of physical appearance and function, affecting self-concept, social interactions, and psychological well-being (Wang et al., 2023). Research in China has focused largely on descriptions of body dissatisfaction, with limited investigations of the underlying psychological mechanisms.

Physical exercise enhances both physical health and psychological well-being. Previous studies suggest associations between consistent exercise participation and positive body perceptions, greater perceived physical competence, and improved self-evaluation (Gualdi-Russo et al., 2022; Li et al., 2025a, 2025b). However, exercise participation among overweight Chinese college students remains relatively low. Despite earlier investigations of the relationship between exercise behavior and body image, the psychological processes linking exercise persistence and body image remain unclear, particularly the roles of social communication and internal evaluations.

Two psychological factors, fat talk and body self-esteem, may influence this relationship. Fat talk refers to negative conversations among peers about body weight or appearance, potentially leading to body dissatisfaction and self-stigma (Wang et al., 2022). Body self-esteem represents self-evaluations of body appearance and functionality, and is linked with body image (Saylan and Soyyiğit, 2022). Previous studies indicate that frequent fat talk leads to negative body perceptions.

There are several theoretical perspectives on these relationships. Self-presentation theory emphasizes the management of social image during interpersonal interactions, potentially influencing body-related behaviors. Social comparison theory suggests body evaluation through comparisons with others, often leading to dissatisfaction where thinness is socially valued. Body self-esteem models highlight the shaping of body image by internal body evaluation. Based on these perspectives, this study examines the relationship between exercise persistence and body image among overweight Chinese college students and explores the mediating roles of fat talk and body self-esteem using a chained mediation model.

## 2 Literature review and research hypotheses

### 2.1 Exercise persistence and body image

Exercise persistence is the stability and commitment with which individuals engage in physical exercise over time and contributes to physical health and psychological well-being (Yin et al., 2023). Body image represents a cognitive and emotional evaluation of physical appearance and bodily characteristics and is linked to self-esteem, social interaction, and mental health (Wang et al., 2023).

It has been suggested that regular physical exercise is associated with more positive body evaluations and perceptions of physical competence among college students (Salvador et al., 2026). However, studies on overweight individuals indicate variability in this relationship according to social and psychological contexts. For example, exercising in certain environments may intensify awareness of body differences, adversely affecting body satisfaction (Alberga et al., 2019).

In terms of theoretical perspectives, self-presentation theory suggests the management of social image during interpersonal interactions, while social comparison theory stresses comparisons with socially valued appearance standards. Body self-identity perspectives emphasize the role of internal identification in shaping body evaluations (de Vaate et al., 2020; MacIntyre et al., 2020; Kling et al., 2018). Participation in exercise by overweight individuals may increase body evaluation, especially in situations that emphasize appearance. Overall, research suggests that exercise persistence is related to body image among overweight individuals, although the strength and direction of this relationship may depend on psychological and social factors.

### 2.2 Mediating role of fat talk

Fat talk refers to negative comments or comparisons about one’s own or others’ body weight and appearance during social interactions and is common among young adults (Kennedy et al., 2023). This increases body awareness and perceived discrepancies between one’s actual and ideal body.

Previous studies suggest that fat talk is linked to body dissatisfaction. For example, individuals who frequently engage in fat talk report lower perceptions of physical attractiveness and higher body-image anxiety (Godoy-Izquierdo et al., 2020; Radwan et al., 2019). In terms of social comparison theory, fat talk encourages body comparisons with socially valued appearance standards, potentially influencing body perception and exercise behavior (Robinson et al., 2020). Thus, fat talk may function as a social communication context in which body-related evaluations and behavioral responses, such as exercise participation, are discussed and reinforced.

However, fat talk may have other effects. Supportive peer contexts may encourage healthy behaviors and provide mutual support (Mills et al., 2021). Therefore, fat talk represents a social interaction pattern influencing body perception and evaluation. Taken together, fat talk is associated with both exercise-related behaviors and body image outcomes, suggesting that it may be a potential mechanism linking exercise persistence and body image in overweight college students.

### 2.3 Mediating role of body self-esteem

Body self-esteem refers to evaluative perceptions of appearance and physical functioning and is an important part of body image. This has been demonstrated in overweight college students (Merino et al., 2024). Greater body self-esteem is linked to more positive body perceptions and vice versa (Lu et al., 2025).

Theoretically, body self-esteem can be understood through body self-identity frameworks, which associate body evaluation with overall self-concept. Exercise behavior may also be related to body self-esteem. Participation in regular physical activity has been associated with more positive perceptions of physical capability, potentially contributing to more favorable body evaluations (Shu et al., 2023). In addition, improved physical ability and fitness may strengthen self-confidence and body self-esteem, especially among overweight individuals (Latino et al., 2021). Taken together, body self-esteem is suggested to be related to both exercise behavior and body image. Therefore, body self-esteem may represent a pathway linking exercise persistence and body image.

### 2.4 Chain mediation effect of fat talk and body self-esteem

Fat talk and body self-esteem may be sequential factors linking exercise persistence and body image. Fat talk leads to body evaluation (Mills et al., 2019) and may increase body-related comparisons, potentially leading to reduced body self-esteem and negative body perceptions (Choi et al., 2021).

Social comparison theory posits that appearance-focused conversations may encourage body comparisons with socially valued standards, thereby influencing self-evaluation and body satisfaction. Furthermore, body self-identity frameworks suggest that individuals construct their body-related self-concept through bodily experiences and self-monitoring during physical activity (Palmeira et al., 2009). Regular exercise may induce greater awareness of physical capability, more positive body evaluations, and reduced body-related anxiety. A decrease in negative appearance-related discussions, together with improved body self-esteem, may improve body-image perceptions. These findings imply that fat talk and body self-esteem may sequentially influence the relationship between exercise persistence and body image.

### 2.5 Research hypotheses

This study proposes the following hypotheses:

*H1*: Exercise persistence is associated with body image among overweight college students.

*H2*: Fat talk mediates the relationship between exercise persistence and body image.

*H3*: Body self-esteem mediates the relationship between exercise persistence and body image.

*H4*: Fat talk and body self-esteem sequentially mediate the relationship between exercise persistence and body image.

**Figure 1:**
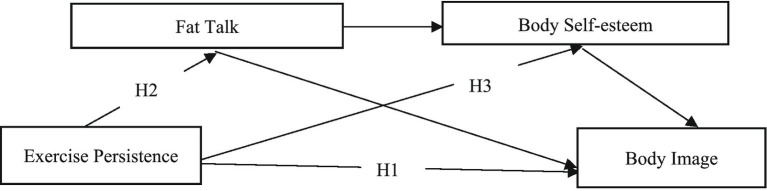
Research framework diagram.

A conceptual model based on these hypotheses is proposed in which body image is influenced directly by exercise persistence and indirectly through fat talk and body self-esteem (Figure 1).

## 3 Study participants and methods

### 3.1 Participants

Overweight students were recruited from several universities in China using a convenience sampling strategy. Overweight was defined according to the Chinese BMI classification standard (BMI ≥ 24 kg/m^2^). Height and weight were measured by trained investigators using standardized equipment before questionnaire administration. Participants were eligible if they were full-time students who regularly attended physical education classes. Individuals with diagnosed metabolic diseases, severe physical disabilities, or other medical conditions restricting physical activity were excluded to ensure the accuracy of overweight classification.

Sample size was determined according to structural equation modeling (SEM) requirements and statistical power analysis. Following Hair et al. (2020), each observed variable requires approximately 10–20 participants, suggesting a minimum sample size of 400–600. G*Power 3.1 was used for power analysis. With *α* = 0.05, statistical power = 0.80, and a medium effect size (*d* = 0.30), the recommended sample size ranged between 684 and 782 participants. Overall, 783 questionnaires were distributed during physical education classes using standardized instructions. Questionnaires with missing responses, logical inconsistencies, or failure to meet inclusion criteria were excluded, resulting in 750 valid questionnaires (valid response rate = 95.79%). Participants were aged 17–25 years (M = 20.66, SD = 1.87), including 359 males (47.87%) and 391 females (52.13%).

The study complied with the ethical principles of the Declaration of Helsinki and was approved by the Ethics Review Committee of Zhaoqing University (Approval No.: 2025073). Written informed consent was obtained from all participants.

### 3.2 Measures

(1) Exercise persistence

Exercise persistence was assessed using the Exercise Persistence Scale developed for Chinese university students (Wang et al., 2016). The scale contains 14 items covering three dimensions: effort commitment, emotional experience, and behavioral habit. Responses were rated on a five-point Likert scale ranging from 1 (strongly disagree) to 5 (strongly agree), with higher scores indicating stronger exercise persistence. The Cronbach’s *α* was 0.943.

(2) Fat talk

Fat talk was measured using the Fat Talk Questionnaire (FTQ) developed by Royal et al. (2013). The instrument includes 14 items assessing the frequency of weight-related negative conversations among peers. Items are rated on a five-point Likert scale from 1 (never) to 5 (always). Higher scores indicate more frequent fat-talk behaviors. The Cronbach’s *α* was 0.932.

(3) Physical self-perception

Physical self-perception was measured using the Chinese revised version of the Physical Self-Perception Profile (PSPP) (Yang and Wu, 2020; Fox and Corbin, 1989). The scale consists of 30 items assessing physical self-worth, athletic ability, physical condition, physical attractiveness, and physical fitness. Items are rated on a four-point scale, with higher scores indicating higher levels of physical self-esteem. The Cronbach’s *α* was 0.930.

(4) Body image

Body image satisfaction was assessed using the Body Image Satisfaction Scale (Andersen and LeGrand, 1991). The scale contains 12 items measuring satisfaction with body parts and overall body characteristics. Responses are rated on a five-point Likert scale ranging from 1 (very dissatisfied) to 5 (very satisfied). Higher scores represent greater body satisfaction. The Cronbach’s *α* was 0.962.

### 3.3 Statistical analysis

Data were analyzed using SPSS 21.0. Descriptive statistics were calculated for demographic variables and study measures. Pearson correlation analysis was performed to examine associations among the main variables. Common method bias was assessed using Harman’s single-factor test. Mediation effects were examined using Model 6 of the PROCESS macro developed by Hayes with 5,000 bootstrap resamples. Statistical significance was recognized at *p* < 0.05.

## 4 Results

### 4.1 Descriptive statistics and group comparisons

Independent sample t-tests and one-way ANOVA were conducted to examine gender and age differences in exercise persistence, body image, fat talk, and body self-esteem. Effect sizes were reported using Cohen’s *d* for *t*-tests and *η*^2^ for ANOVA. The descriptive statistics and group comparison results are presented in Tables 1, 2.

**Table 1:** Gender differences in exercise persistence, body image, fat talk, and body self-esteem.

Variable	Gender	Number (%)	M	SD	*t*	*p*
Exercise persistence	Male	359(47.87%)	57.983	8.586	−1.232	0.218
	Female	391 (52.13%)	58.726	7.934		
	Total	750	58.371	8.255		
Body image	Male	359 (47.87%)	80.159	10.272	−1.454	0.146
	Female	391 (52.13%)	81.194	9.228		
	Total	750	80.699	9.749		
Fat talk	Male	359 (47.87%)	30.398	8.219	−0.127	0.899
	Female	391 (52.13%)	30.473	7.873		
	Total	750	30.437	8.035		
Body self-esteem	Male	359 (47.87%)	74.763	12.520	−0.227	0.821
	Female	391 (52.13%)	74.962	11.452		
	Total	750	74.867	11.968		

**Table 2:** Age differences in exercise persistence, body image, fat talk, and body self-esteem.

Variable	Age	Number (%)	M	SD	*F*	*p*
Exercise persistence	17	40 (5.33%)	58.150	7.744	2.023	0.04
	18	67 (8.93%)	60.642	7.225		
	19	109 (14.53%)	57.982	9.033		
	20	136 (18.13%)	57.044	9.508		
	21	157 (20.93%)	58.248	7.636		
	22	107 (14.27%)	58.944	7.444		
	23	70 (9.33%)	57.100	8.370		
	24	41 (5.47%)	61.244	5.594		
	25	23 (3.07%)	58.739	9.752		
	Total	750	58.371	8.255		
Body image	17	40 (5.33%)	80.500	8.231	0.724	0.67
	18	67 (8.93%)	80.254	10.046		
	19	109 (14.53%)	79.982	10.200		
	20	136 (18.13%)	80.360	10.662		
	21	157 (20.93%)	81.096	9.791		
	22	107 (14.27%)	80.589	9.695		
	23	70 (9.33%)	80.071	9.383		
	24	41 (5.47%)	83.683	8.026		
	25	23 (3.07%)	82.130	7.313		
	Total	750	80.699	9.749		
Fat talk	17	40 (5.33%)	33.075	6.354	1.14	0.334
	18	67 (8.93%)	30.134	8.937		
	19	109 (14.53%)	30.761	8.332		
	20	136 (18.13%)	29.787	8.529		
	21	157 (20.93%)	30.586	7.941		
	22	107 (14.27%)	30.579	8.178		
	23	70 (9.33%)	30.800	6.381		
	24	41 (5.47%)	28.122	7.804		
	25	23 (3.07%)	30.391	8.055		
	Total	750	30.437	8.035		
Body self-esteem	17	40 (5.33%)	73.300	10.371	0.743	0.653
	18	67 (8.93%)	77.015	13.099		
	19	109 (14.53%)	74.303	13.256		
	20	136 (18.13%)	73.750	12.634		
	21	157 (20.93%)	75.146	10.924		
	22	107 (14.27%)	74.738	11.448		
	23	70 (9.33%)	74.629	10.890		
	24	41 (5.47%)	76.927	12.364		
	25	23 (3.07%)	76.348	12.737		
	Total	750	74.867	11.968		

No significant differences were found between male and female participants across the four variables (*p* > 0.05). Male participants reported slightly lower scores in exercise persistence (M = 57.98, SD = 8.59) compared with females (M = 58.73, SD = 7.93), *t*(748) = −1.232, *p* = 0.218, *d* = 0.09. Similarly, males reported slightly lower scores in body image (M = 80.16, SD = 10.27) than females (M = 81.19, SD = 9.23), *t*(748) = −1.454, *p* = 0.146, *d* = 0.10. No significant gender differences were observed for fat talk, *t*(748) = −0.127, *p* = 0.899, *d* = 0.01, or body self-esteem, *t*(748) = −0.227, *p* = 0.821, *d* = 0.02. Overall, the effect sizes for gender differences were small.

Significant differences in exercise persistence were found across age groups, *F*(8, 741) = 2.023, *p* = 0.040, *η*^2^ = 0.02. However, no significant age differences were found for body image, *F*(8, 741) = 0.724, *p* = 0.670, *η*^2^ = 0.01, fat talk, *F*(8, 741) = 1.140, *p* = 0.334, *η*^2^ = 0.01, or body self-esteem, *F*(8, 741) = 0.743, *p* = 0.653, *η*^2^ = 0.01.

As multiple group comparisons were conducted, the possibility of Type I error should be acknowledged. No formal adjustment for multiple comparisons (e.g., Bonferroni correction) was applied; therefore, these exploratory comparisons should be interpreted with caution.

### 4.2 Common method bias test

Potential common method bias was assessed using Harman’s single-factor test with exploratory factor analysis. Five factors with eigenvalues >1 were identified. The first factor accounted for 13.909% of the total variance, which is below the 40% threshold, indicating that common method bias was not a major concern.

### 4.3 Correlation analysis of exercise persistence, body image, fat talk, and body self-esteem

The Pearson correlation matrix is presented in Table 3.

**Table 3:** Correlations between exercise persistence, body image, fat talk, and body self-esteem.

Variables	M ± SD	Exercise persistence	Body image	Fat talk	Body self-esteem
Exercise persistence	58.371 ± 8.255	1			
Body image	80.699 ± 9.749	0.628**	1		
Fat talk	30.437 ± 8.035	−0.522**	−0.572**	1	
Body self-esteem	74.867 ± 11.968	0.659**	0.690**	−0.563**	1

*N* = 750. ** *p* < 0.01 (two-tailed).

Exercise persistence was positively correlated with body image (*r* = 0.628, *p* < 0.01) and body self-esteem (*r* = 0.659, *p* < 0.01) and negatively correlated with fat talk (*r* = −0.522, *p* < 0.01). Body image was positively associated with body self-esteem (*r* = 0.690, *p* < 0.01) and negatively with fat talk (*r* = −0.572, *p* < 0.01). Furthermore, fat talk was negatively correlated with body self-esteem (*r* = −0.563, *p* < 0.01).

### 4.4 Chain mediation analysis

**Figure 2:**
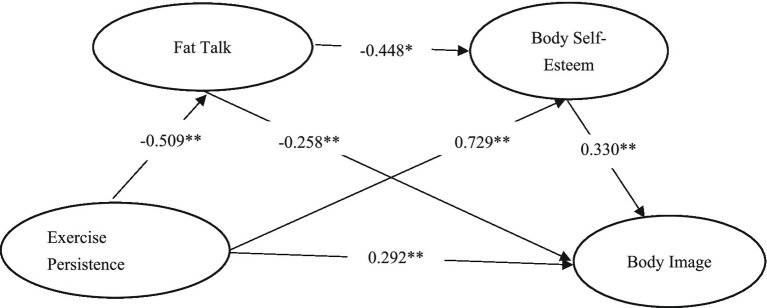
Chain mediation model of fat talk and body self-esteem on exercise persistence and body image.

The chain mediation analysis used exercise persistence as the independent variable, body image as the dependent variable, and fat talk and body self-esteem as sequential mediators. Bootstrap estimation with 5,000 resamples was used to calculate regression coefficients and 95% confidence intervals (Table 4; Figure 2).

**Table 4:** Regression analysis of the chain mediation model between exercise persistence and body image among overweight college students.

Variables	Fat talk		Body self-esteem		Body image		Overall effect	
	*β*	*t*	*β*	*t*	*β*	*t*	*β*	*t*
Exercise persistence	−0.509	−16.978**	0.729	16.014**	0.292	6.386**	0.740	20.405**
Obesity discussions			−0.448	−9.847**	−0.258	−6.938**		
Body self-esteem					0.330	11.251**		
*R* ^2^	0.276		0.501		0.561		0.398	
*F*	98.609**		185.312**		165.851718**		139.006**	

** *p* < 0.001.

Regression analysis indicated that exercise persistence was significantly associated with body image (*β* = 0.740, *t* = 20.405, *p* < 0.001). When the mediators were included in the model, the direct association between exercise persistence and body image remained significant (*β* = 0.292, *t* = 6.386, *p* < 0.001). Exercise persistence was negatively associated with fat talk (*β* = −0.509, *t* = −16.978, *p* < 0.001), while fat talk was negatively associated with body image (*β* = −0.0258, *t* = −6.938, *p* < 0.001). Exercise persistence was positively associated with body self-esteem (*β* = 0.729, *t* = 16.014, *p* < 0.001), and body self-esteem was positively associated with body image (*β* = 0.0330, *t* = 11.251, *p* < 0.001). In addition, fat talk was negatively linked to body self-esteem (*β* = −0.448, *t* = −9.847, *p* < 0.001).

All regression paths in the model were significant (*p* < 0.001). The detailed results of the chain mediation model are presented in Table 4.

Bootstrap analyses were performed to examine the indirect effects of the mediation model (Table 5). The total indirect effect of exercise persistence on body image was significant (effect = 0.447, boot SE = 0.033, 95% CI [0.389, 0.516]), indicating indirect associations through mediating variables. Three indirect pathways were identified. The first through fat talk (exercise persistence → fat talk → body image) showed a significant effect (effect = 0.131, boot SE = 0.021, 95% CI [0.091, 0.178]). The second through body self-esteem (exercise persistence → body self-esteem → body image) was also significant (effect = 0.241, boot SE = 0.035, 95% CI [0.194, 0.380]). In addition, the sequential pathway through both mediators (exercise persistence → fat talk → body self-esteem → body image) demonstrated a significant indirect effect (effect = 0.075, boot SE = 0.026, 95% CI [0.011, 0.098]). None of the confidence intervals included zero, indicating that all were significant (Table 5).

**Table 5:** Bootstrap results for the chain mediation model.

Effect type	Efficiency value	Boot SE	Bootstrap 95% CI		Effect proportion
			Lower limit	Upper bound	
Total effect	0.740	0.036	0.669	0.811	100%
Direct effect	0.292	0.046	0.203	0.382	39.46%
Exercise persistence → fat talk → body image	0.131	0.021	0.091	0.178	17.70%
Exercise persistence → body self-esteem → body image	0.241	0.350	0.194	0.380	32.57%
Exercise persistence → fat talk → body self-esteem → body image	0.075	0.026	0.011	0.098	10.14%
Total indirect effect	0.447	0.033	0.389	0.516	60.41%

## 5 Discussion

This study examined the relationships among exercise persistence, fat talk, body self-esteem, and body image among overweight college students. Exercise persistence was positively associated with body image, with fat talk and body self-esteem mediating this relationship, both independently and sequentially. Specifically, lower levels of fat talk and higher levels of body self-esteem were associated with more positive body image evaluations.

### 5.1 Exercise persistence and body image

Exercise persistence was positively associated with body image (*r* = 0.628, *p* < 0.01).

Theoretically, this association may be understood within the framework of self-presentation theory and social comparison theory. Individuals regulate their behavior in social contexts to create favorable impressions (de Vaate et al., 2020). For overweight college students, physical activity improve perceived physical appearance and competence, leading to positive body evaluations. This is consistent with previous findings (Yang et al., 2024).

Social comparisons may also explain the observed relationship. In university environments, students frequently compare physical appearances. Improved physical condition may thus lead to positive body evaluations (Shang et al., 2021; Radwan et al., 2019). The findings may also be interpreted in terms of body self-identity theory, where body perception contributes significantly to self-concept. Thus, sustained exercise participation was significantly linked to more positive body image evaluations among overweight students.

Group comparisons indicated no significant gender differences in exercise persistence, body image, fat talk, or body self-esteem (*p* > 0.05). In contemporary university environments, male and female students often share physical education settings and health-related goals, which may contribute to comparable exercise participation and body evaluation. Taken together, the findings indicate an association between exercise persistence and body image among overweight college students. However, the cross-sectional nature of the study precludes conclusions regarding causal relationships.

### 5.2 Mediating role of fat talk

The mediation analysis indicated that fat talk mediated the relationship between exercise persistence and body image. Specifically, the indirect pathway through fat talk was significant (effect = 0.131, 95% CI [0.091, 0.178]), suggesting that exercise persistence was associated with body image through changes in fat-talk engagement. This finding provides empirical support for the proposed mediating pathway.

Previous research shows that higher levels of fat talk are associated with lower body satisfaction and body image levels. In social contexts such as university campuses, comparisons with perceived peer standards are common (Mills et al., 2019; Mills et al., 2021; Radwan et al., 2019).

In this study, exercise persistence was negatively associated with fat talk (*β* = −0.509, *p* < 0.001), while fat talk was negatively associated with body image (*β* = −0.0258, *p* < 0.001). These relationships suggest that students with greater exercise persistence reported lower levels of fat talk, in turn leading to more positive body image evaluations. This is consistent with findings suggesting links between regular physical activity and positive body-related perceptions and reduced fat talk. The findings indicate that fat talk functions as a link between exercise behavior and body perception.

Overall, the results suggest that exercise persistence is associated with body image through an indirect pathway involving fat talk. However, given the cross-sectional nature of the data, these findings should be interpreted as associations rather than causal relationships.

### 5.3 Mediating role of body self-esteem

The mediation analysis indicated that body self-esteem significantly and indirectly mediated the relationship between exercise persistence and body image (effect = 0.241, 95% CI [0.194, 0.380]). Exercise persistence was positively associated with body self-esteem (*β* = 0.729, *p* < 0.001), and body self-esteem was positively associated with body image (*β* = 0.330, *p* < 0.001). These results suggest that greater exercise persistence is linked with higher body self-esteem, and thereby more positive body image evaluations. Body self-esteem is an important component of individuals’ evaluations of their physical appearance, body shape, and physical competence (Merino et al., 2024; Shang et al., 2021). Individuals with higher body self-esteem tend to evaluate their bodies more positively and may place greater emphasis on physical functioning and health. Therefore, body self-esteem is associated with body image perception.

From a theoretical perspective, body self-identity theory suggests that body perception is an important component of self-concept. Positive perceptions of body capability and appearance may lead to stronger body-related self-evaluations and more positive body image assessments. In this study, sustained engagement in exercise was significantly associated with higher body self-esteem among overweight college students, consistent with previous reports (Shang et al., 2021). Therefore, body self-esteem may represent an important psychological pathway linking exercise persistence and body image.

Overall, the findings indicate that exercise persistence is associated with body image through an indirect pathway involving body self-esteem. However, the cross-sectional data preclude the drawing of conclusions as to cause.

### 5.4 Chain mediation effect of fat talk and body self-esteem

The results further indicated a significant indirect chain mediation pathway involving fat talk and body self-esteem in the relationship between exercise persistence and body image (effect = 0.075, 95% CI [0.011, 0.098]). This finding suggests that exercise persistence was associated with body image through a sequential pathway in which lower levels of fat talk were related to higher body self-esteem and leading to more positive body image evaluations. Specifically, exercise persistence was negatively associated with fat talk (*β* = −0.509, *p* < 0.001), fat talk was negatively associated with body self-esteem (*β* = −0.448, *p* < 0.001), and body self-esteem was positively associated with body image (*β* = 0.330, *p* < 0.001). These associations indicate that students with greater exercise persistence tended to report lower levels of fat talk and higher levels of body self-esteem, which were in turn associated with more positive body image evaluations.

Previous studies have shown that weight-related conversations and negative appearance-focused discussions are associated with lower body satisfaction and body-related self-evaluations among university students (Mills et al., 2019; Koçak et al., 2026). Within social contexts where body appearance is frequently discussed, exposure to negative weight-related discourse may lead to increased body-focused social comparison and reduced body self-esteem. Lower levels of body self-esteem have also been consistently linked to less positive body-image perceptions. The present findings extend these observations by suggesting that fat talk and body self-esteem may operate sequentially as interpersonal and psychological mechanisms associated with body image evaluations among overweight college students. In this model, fat talk reflects an interpersonal communication context related to body evaluation, while body self-esteem represents an internal psychological evaluation of the body.

Overall, the results suggest that exercise persistence is associated with body image through multiple indirect pathways, including the sequential pathway involving fat talk and body self-esteem. However, the cross-sectional design of the study precludes the drawing of conclusions regarding causality.

## 6 Practical significance

The findings of this study have practical implications for promoting the physical and psychological well-being of overweight college students. The results suggest that exercise persistence is associated with more positive body image, through lower levels of fat talk and higher levels of body self-esteem. These findings highlight the importance of supportive exercise environments and positive body-related communication within university physical education contexts.

From a practical perspective, universities should encourage sustained participation in physical activity while fostering respectful and non-stigmatizing body-related discussions on campus. Physical education instructors and health educators can also emphasize positive body feedback and supportive peer interactions to assist in the development of healthier body perceptions. Overall, these findings offer practical insights for integrating physical activity promotion with psychological well-being in university health education programs.

## 7 Research limitations and future directions

The study has several limitations. First, although the sample size was relatively large (*n* = 750), participants were recruited from selected universities in China, primarily comprehensive public institutions located in different regions, which may limit the representativeness of the sample, particularly for students from vocational or private universities.

Second, the cross-sectional design restricts the ability to infer causal relationships among exercise persistence, fat talk, body self-esteem, and body image. Future studies should use longitudinal or experimental designs to clarify the temporal relationships among these variables.

Third, all variables were measured using self-reported questionnaires, which may be influenced by social desirability bias. To reduce this risk, the survey was conducted anonymously.

Finally, this study focused on fat talk and body self-esteem as mediating variables. Future research could consider additional psychological or social factors to further explore the mechanisms linking exercise behavior and body image.

## 8 Conclusion

Based on empirical data of overweight college students in China, this study examined the relationships among exercise persistence, fat talk, body self-esteem, and body image. The results showed that exercise persistence was positively associated with body image. In addition, fat talk and body self-esteem were found to mediate this relationship, both independently and sequentially. Specifically, lower levels of fat talk and higher levels of body self-esteem were associated with more positive body image evaluations.

These findings contribute to the understanding of physical activity and psychological well-being by highlighting the interpersonal and psychological pathways linking exercise persistence and body image. From a practical perspective, the results suggest that university health-promotion programs may benefit from encouraging sustained exercise participation while also addressing negative body-related conversations and supporting positive body self-esteem. These efforts may contribute to healthier body perceptions and more supportive campus environments.

## Data Availability

The raw data supporting the conclusions of this article will be made available by the authors, without undue reservation.
