# Supplementary material for: Relationship between exercise persistence and body image among overweight Chinese college students: the mediating role of fat talk and body self-esteem
Source: Front Psychol. 2026 Jun 4;17:1824393. doi: 10.3389/fpsyg.2026.1824393 (PMC13275279; doi:10.3389/fpsyg.2026.1824393)
Supplement: Supplementary file 1 [file Supplementary_file_1.docx]

**X: Independent Variable: Exercise Persistence**

Exercise Persistence Scale Scoring and Specific Items

(Likert 5-point scale: Strongly Disagree 1 point, Disagree 2 points, Neutral 3 points, Agree 4 points, Strongly Agree 5 points; total score 1–5 points)

1. Each time I engage in physical activity, it lasts at least one hour.

2. I have been consistently exercising for at least six months.

3. I engage in physical exercise at least 3 times per week.

4. I habitually exercise when it's time to exercise.

5. I strive to practice new skills to improve myself.

6. Regardless of whether I enjoy the exercise, I always give my best effort each time.

7. I am determined to stick with my exercise routine.

8. I am willing to set aside relatively fixed times to maintain my exercise routine.

9. I try to eliminate distractions to stick with my exercise routine.

10. I enjoy the feeling physical exercise brings.

11. I like exercising with others.

12. I often feel uplifted after exercising.

13. I often feel stronger after exercising.

14. I feel physically and mentally refreshed after exercising.

**Y: Dependent Variable: Body Image**

Please select a number that best reflects your recent physical activity level; numbers 1-5 indicate agreement level (1=Strongly Disagree, 2=Somewhat Disagree, 3=Unsure, 4=Somewhat Agree, 5=Strongly Agree). Mark the option that matches your actual situation with a “√”. All questions are single-choice with no right or wrong answers—please complete all sections. Thank you!

Strongly disagree = 1 point, Disagree = 2, Unsure = 3, Agree = 4, Strongly agree = 5

1. I feel my body is natural.
2. 2. I feel my body is healthy.

3. I feel my body is whole.

4. I am confident I can make myself stronger.

5. I feel I can control some aspects of my health.

6. I cannot control the factors that determine my health.

7. I feel energetic.

8. I feel physically comfortable.

9. My physical strength allows me to do everything I want to do.

10. I feel uncomfortable or embarrassed because of my poor physical condition.

11. My poor physical condition prevents me from doing what I want to do.

12. I am physically strong.

13. I have limited my social activities due to physical symptoms caused by disease treatment (surgery, chemotherapy, radiation therapy), such as nausea/vomiting, fatigue, pain, etc.

14. I feel uncomfortable or embarrassed due to physical symptoms caused by disease treatment (surgery, chemotherapy, radiation therapy), such as nausea/vomiting, fatigue, pain, etc.

15. Physical symptoms caused by disease treatment (surgery, chemotherapy, radiation therapy), such as nausea/vomiting, fatigue, pain, etc., prevent me from doing what I want to do.

16. I feel comfortable with my physical appearance.

17. I feel uncomfortable or embarrassed by my physical appearance.

18. I have limited my social activities because of my physical appearance.

**M1: Mediating Variable 1: Discussions About Weight**

Instruction: When you are with one or more close female friends, how often do you engage in the following behaviors?

Never = 1 point, Rarely = 2 points, Sometimes = 3 points, Often = 4 points, Always = 5 points.

1. When I am with one or more close female friends, I complain about my flabby arms.

2 When I'm with one or more close female friends, I complain about having too much belly fat.

3 When I'm with one or more close female friends, I envy female celebrities with good figures in the media and complain about my own body shape.

4 When I'm with one or more close female friends, I complain about my poor body proportions. 1 2 3 4 5

5 When I'm with one or more close female friends, I complain that I hate my entire body.

6 When I'm with one or more close female friends, I complain that I'm fat.

7 When I'm with one or more close female friends, I complain that I shouldn't eat foods that make me fat.

8 When I'm with one or more close female friends, I complain that I've gained weight.

9 When I'm with one or more close female friends, I complain that my clothes are too tight.

10 When I'm with one or more close female friends, I complain that I need to stop eating so much.

11 When I'm with one or more close female friends, I criticize my own or my friends' figures.

12 When I'm with one or more close female friends, I complain about feeling pressured to lose weight. 13 When I'm with one or more close female friends, I complain that my body disgusts me.

14 When I'm with one or more close female friends, I complain that my figure isn't standard.

M2: Mediating Variable 2: Measurement of Body Self-Esteem

Body Self-Esteem Scale

What kind of person am I? Below are statements describing people. There are no right or wrong answers.

Please select the option that best fits you from the four choices for each statement.

“Not at all true” = 1 point, “Somewhat untrue” = 2 points, “Somewhat true” = 3 points, “Completely true” = 4 points

1. I feel capable of excelling at any athletic activity.

2. I always believe I can maintain excellent physical condition and fitness.

3. Compared to most people, I have an attractive body.

4. My body is much stronger than most people of the same gender.

5. Sometimes I feel incredibly proud of my physique and physical abilities.

6. When it comes to athletic ability, I am among the best.

7. I regularly participate in vigorous physical exercise.

8. I find it easy to keep my body attractive.

9. Compared to most people of my gender, I run faster.

10. I am satisfied with my physical condition or fitness level.

11. I feel very confident when participating in sports.

12. Compared to most people, I always have better energy and stamina.

13. I feel very comfortable wearing minimal clothing.

14. When it comes to physical coordination, I am always confident.

15. I feel completely confident about my physical appearance.

16. I am among the best performers when participating in sports.

17. I consistently feel confident and at ease in fitness and exercise settings.

18. Others often envy me for my outstanding physique or figure.

19. I feel highly confident regarding my physical strength.

20. I consistently have positive experiences with my body, such as satisfaction.

21. I'm usually among the first to learn new movements.

22. I feel very confident in my ability to maintain regular exercise and physical fitness.

23. My physique looks better than most people's.

24. My explosive power is significantly better than most people of the same gender.

25. I always take extra care of my body.

26. When given the chance, I'm always the first to participate in sports.

27. Compared to most people, I consistently maintain a higher level of physical health.

28. I feel completely confident about my physical appearance.

29. In situations requiring physical strength, I feel I'm among the best.

30. I'm very satisfied with the type of body I have.
